# A Prospective Clinical Trial of Efgartigimod for New-Onset Generalized Myasthenia Gravis

**DOI:** 10.2174/011570159X366227250716054651

**Published:** 2025-08-06

**Authors:** Chi Ma, Jingyi Shen, Ying Zhu, Ruixia Zhu

**Affiliations:** 1 Department of Neurology, The First Hospital, China Medical University, Shenyang, China

**Keywords:** Myasthenia gravis, anti-cholinergic receptor, immunosuppressive therapy, new-onset AChR-gMG, Efgartigimod, corticosteroids

## Abstract

**Introduction:**

Numerous studies have demonstrated that efgartigimod is effective in treating myasthenia gravis (MG) across various patient populations. However, there is limited evidence regarding its use in patients with new-onset acetylcholine receptor antibody-positive generalized MG (AChR-gMG). Therefore, this study aimed to investigate the real-world safety and effectiveness of efgartigimod in Chinese patients with new-onset anti-cholinergic receptor (AChR)- gMG.

**Methods:**

This prospective study was conducted in 29 patients with new-onset AChR-gMG, with a three-month follow-up. The Myasthenia Gravis Activities of Daily Living (MG-ADL) score, Quantitative Myasthenia Gravis score, prednisone dose, laboratory data, and adverse events were assessed at every follow-up visit.

**Results:**

At 4, 8, and 12 weeks, the mean change in MG-ADL scores was 8.13 ± 3.66, 7.41 ± 4.22, and 6.37 ± 4.67, respectively. Compared with the baseline, 96% (28/29) of patients achieved an MG-ADL response (defined as a decrease of ≥2 points), with a mean response time of 0.81 ± 0.53 weeks (5.67 ± 3.71 days). After one cycle, 52% (15/29) of patients achieved minimal symptom expression (MSE), while 41% maintained MSE at 12 weeks. Moreover, 89% and 72% of MG-ADL responders sustained for 8 and 12 consecutive weeks, respectively. Additionally, patients with thymomatous MG exhibited a poorer response to efgartigimod and required two infusion cycles. All patients were able to reduce their daily steroid dose, and the mean daily prednisone dose decreased by 10.73 mg per day. The treatment was well tolerated, and a few mild adverse events were reported.

**Discussion:**

These results demonstrate the clinical significance of efgartigimod in patients with new-onset AChR-gMG, achieving rapid symptom relief and steroid reduction. Additionally, the potential of efgartigimod to serve as a bridge treatment, facilitating a steady transition to long-term conventional immunosuppressive therapy, was demonstrated. Due to limitations in this study, such as a small sample size, larger randomized controlled trials are needed to validate.

**Conclusion:**

Our study showed that efgartigimod is clinically beneficial and offers rapid symptom control in patients with new-onset AChR-gMG. A more aggressive application of efgartigimod in combination with corticosteroids may lead to a smoother therapeutic transition, which will further maintain favorable conditions.

## 1 INTRODUCTION

Myasthenia gravis (MG) is a rare autoimmune disease that affects the neuromuscular junctions, primarily characterized by muscle weakness and fatigue that worsen after exercise, significantly impairing patients' quality of life. The pathophysiology of MG involves autoantibodies against the acetylcholine receptor (AChR), muscle-specific tyrosine kinase (MuSK), and low-density lipoprotein receptor-related protein 4 (LRP4). Traditionally, the treatment of MG has relied on acetylcholinesterase inhibitors and long-term immunosuppressive therapy with corticosteroids and nonsteroidal immunosuppressants [1]. While these treatments alleviate symptoms in many patients, some do not respond adequately or cannot tolerate the side effects, highlighting the urgent need for new targeted treatments. Recent molecular therapies, including complement inhibitors, neonatal Fc Receptor (FcRn) antagonists, B-cell depleting agents, and chimeric antigen receptor T-cell-based therapies, have updated the MG treatment landscape.

Efgartigimod offers new hope for patients with MG. With its high affinity for FcRn, efgartigimod, the first FcRn antagonist authorized for the treatment of MG, lowers the levels of circulating immunoglobulin G (IgG), including pathogenic autoantibodies, without affecting the levels of other Igs or albumin. In the pivotal phase 3 ADAPT trial, the efficacy of efgartigimod was demonstrated to be significant and rapid, resulting in a reduction in patient burden through symptom improvement [2]. According to multiple studies, real-world experiences with efgartigimod align with the findings of the ADAPT study, demonstrating that efgartigimod works for diverse patient subtypes and can be incorporated into individualized MG treatment strategies. These not only provide data on therapeutic responses and adverse events but also provide insights into dosing schedules for subsequent clinical practice. Despite a growing body of international real-world data supporting the efficacy, tolerability, and safety of efgartigimod, there is a paucity of relevant evidence regarding its use in patients with new-onset AChR antibody-positive generalized MG (AChR-gMG). Owing to China's unique genetic, environmental, and healthcare characteristics, localized studies are necessary to validate these outcomes. Therefore, we conducted a prospective study to investigate the safety and effectiveness of efgartigimod in Chinese patients with new-onset AChR-gMG to explore the most suitable scenario for patients with MG.

## 2 METHODS

### 2.1 Study Design and Patient Selection

This prospective study was conducted at our single MG center and included 29 adult patients with new-onset generalized MG (gMG). All patients were seropositive for the AChR antibody and had received at least one cycle of efgartigimod treatment from September 1, 2023, until April 30, 2024, with a three-month follow-up. Participants were included according to the following criteria: (1) age≥18 years; (2) new-onset gMG; (3) Quantitative Myasthenia Gravis (QMG) score≥6 (>50% non-ocular), evaluated after discontinuation of acetylcholinesterase inhibitors for more than 12 hours; (4) seropositive for AChR antibody; (5) class II, III, or IV MG as per the Myasthenia Gravis Foundation of America (MGFA) classification; and (6) administration of efgartigimod and follow up for at least 12 weeks. New-onset gMG was defined as gMG onset within 12 months with no time limit for isolated ocular symptoms.

Basic clinical information of patients with MG, including sex, age at MG onset, disease duration, comorbidities, and details of any medical treatment administered, was recorded. Patients enrolled in the trial received an intravenous infusion of efgartigimod at a dose of 10 mg/kg over 1 hour, with four weekly infusions administered throughout the cycle. The subsequent cycle was flexible, and initiation depended on the treating physician's discretion, based on the acute exacerbation of the Myasthenia gravis Activities of Daily Living (MG-ADL) score. The regimen for readministration of efgartigimod was the same as that for the first cycle. The baseline prednisone dose was 20–30 mg and did not exceed 60 mg.

The study was approved by the Ethics Committee of the First Affiliated Hospital of China Medical University (Protocol No. 2024-507-2, First Affiliated Hospital of China Medical University). All the participants provided written informed consent.

### 2.2 Clinical Assessment of MG

Comprehensive visits were performed at baseline, as well as at the 1^st^, 2^nd^, 3^rd^, 4^th^, 8^th^, and 12^th^ weeks, and at the final follow-up, which consisted of a full assessment of QMG and MG-ADL at the end of the week. An increase in the QMG score of more than 4 points was considered an exacerbation, whereas clinical improvement was defined as a decrease in the QMG score of more than 3 points. MG-ADL responders showed a 2-point improvement in the MG-ADL score. Minimal symptom expression (MSE) refers to the absence of functional limitations caused by MG with an ADL score (0,1).

### 2.3 Laboratory Investigations and Safety Evaluation

Serum Ig and AChR antibody levels were measured at baseline and week 4. Lipid and albumin profiles were also assessed to monitor treatment-related metabolic effects. The main adverse events of concern that were monitored during follow-up were infection, fever, rash, diarrhea, headache, dizziness, nausea, allergy, and hypertension. Importantly, we observed injection site reactions within a week after the injection, which usually manifest as erythema, swelling, pruritus, and pain at the injection site.

### 2.4 Statistical Analyses

Descriptive statistics were used to summarize the demographic and clinical characteristics of the study population. GraphPad Prism 8.0 and IBM SPSS version 25.0 (SPSS Inc., Chicago, IL, USA) were used for statistical analysis. Changes in clinical scale scores and laboratory parameters were analyzed using paired t-tests to compare pre- and post-treatment values, whereas multiple independent samples were analyzed using the χ2 test. Survival analysis was used to investigate the effectiveness of efgartigimod on the time to reach MSE, and a Kaplan-Meier curve was plotted. Statistical significance was set at *P*<0.05.

## 3 RESULTS

### 3.1 Baseline Clinical Characteristics

All patients completed at least four infusions and 12 weeks of follow-up. Their mean age was 62.24 ± 11.37 years. The majority of patients (69%) were classified as MGFA III at the onset of MG and had mean baseline QMG and MG-ADL scores as high as 13.52 ± 3.64 and 10.38 ± 2.71, respectively, despite having received conventional MG therapy (pyridostigmine (97%), prednisone (69%), and tacrolimus (24%) at the time of enrollment). Specific information is provided in Table **1**.

### 3.2 Clinical Outcomes

**Fig. (1):**
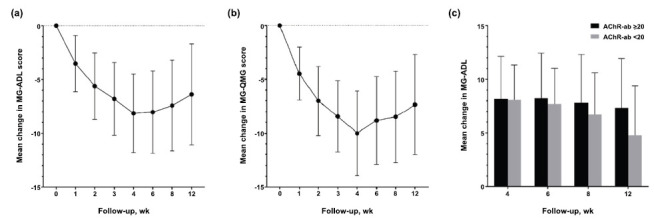
Mean change in MG-ADL scores (**a**), QMG scores (**b**), and mean change in MG-ADL scores in two subgroups (AChR antibody <20 nmol/L and ≥20 nmol/L) (**c**) during the follow-up.

Compared with the baseline, 96% of patients demonstrated an MG-ADL response (ADL ≥ 2 points) after one cycle, and the time to response was 0.81 ± 0.53 weeks (5.67 ± 3.71 days). Changes in MG-ADL were 8.13 ± 3.66, 7.41 ± 4.22, and 6.37 ± 4.67 at weeks 4, 8, and 12 compared with baseline (Fig. **1a**). The changes in QMG score were 10 ± 3.94, 8.48 ± 4.24, and 7.34 ± 4.68 at 4, 8, and 12 weeks, respectively (Fig. **1b**). Clinical improvement was observed in 93% of patients (QMG ≥ 3 points). Furthermore, the patients were divided into two subgroups based on their AChR antibody titers (AChR antibody titer < 20 nmol/L and ≥ 20 nmol/L). The proportions of clinical improvement in the MG-ADL scores were similar between the two subgroups, suggesting that efgartigimod remained effective for gMG with a lower antibody titer, thereby expanding the spectrum of appropriate populations (Fig. **1c**).

**Fig. (2):**
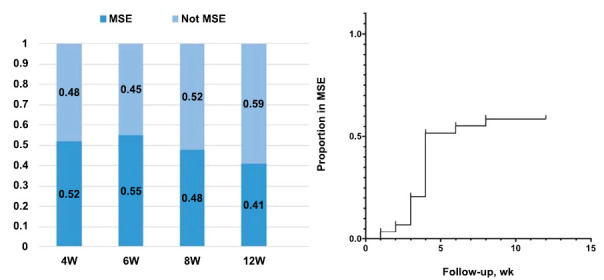
The proportion of patients who did and did not reach MSE at 4, 6, 8, and 12 weeks (**a**), and the proportion of patients who reached MSE during the follow-up (**b**).

**Fig. (3):**
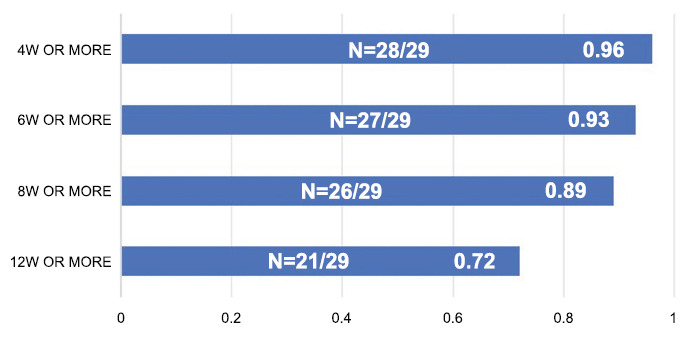
The proportion of MG-ADL responders at 4, 6, 8, and 12 weeks.

After one cycle, 52% (15/29) of the patients achieved MSE, and 41% maintained MSE at week 12 (Figs. **2a**, **b**). For 8 and 12 consecutive weeks, 89% and 72% of the MG-ADL responders, respectively, were sustained. The proportion of persistent MG-ADL responders was higher than that in the ADAPT study at 8 and 12 consecutive weeks, revealing that early active treatment for new-onset MG yielded better clinical outcomes (Fig. **3**).

**Fig. (4):**
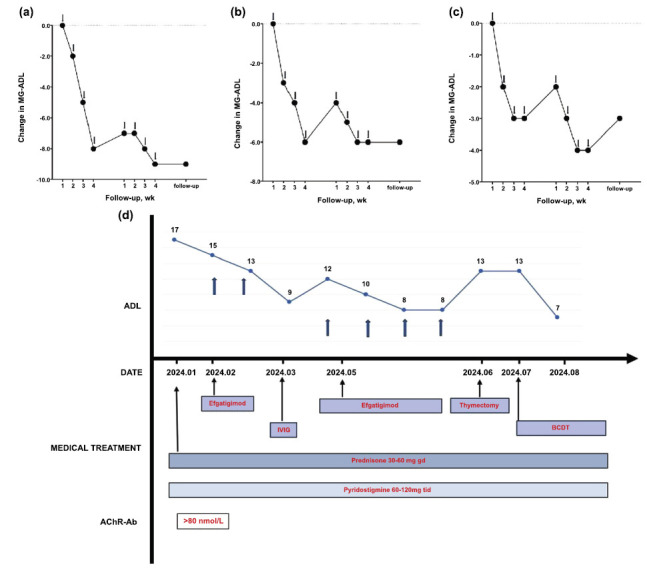
The mean change in MG-ADL scores of patients 1 (**a**), 2 (**b**), 3 (**c**), and 4 (**d**) who received 2 cycles of efgartigimod. BCDT, B cell depletion therapy.

Four patients (4/29) received a second cycle of efgartigimod after an average of 5.2 weeks. These four patients showed clinical improvement in the second round, supporting the fact that efgartigimod retained its effect when re-administered (Figs. **4a**-**d**). Notably, 79% of the patients did not require subsequent cycles. Two patients benefited only from two MG-ADL point changes, which did not represent continuous improvements; therefore, they were switched to other treatments after one cycle.

### 3.3 Steroid-sparing effect of Efgartigimod

The average daily dose of prednisolone was reduced to 20.2 ± 7.72 mg by week 4 and 9.47 ± 5.24 mg by week 12. The mean daily dose of prednisone was decreased by 10.73 mg per day. All patients taking corticosteroids at baseline had their daily doses tapered during the cycling period, illustrating the vital role of efgartigimod in the steroid-sparing effect, with no exacerbation of MG. Only one patient was admitted to the intensive care unit (ICU) and received intravenous Ig (IVIg) for MG exacerbation.

### 3.4 IgG and AChR Antibody Levels

**Fig. (5):**
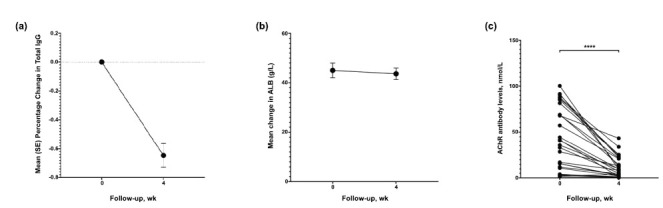
The mean ± standard deviation of IgG (**a**), albumin levels (**b**), and the individual AChR antibody levels (**c**) at baseline and 4 weeks.

After the fourth infusion, IgG levels decreased from 12.35 ± 2.33 g/L to 4.38 ± 1.37 g/L (Fig. **5a**). The mean albumin levels were 45.05 ± 2.94 g/L at baseline and 43.7 ± 2.3 g/L at week 4 (Fig. **5b**). The average albumin, IgA, and IgM levels did not change significantly after efgartigimod infusion. The average AChR antibody levels decreased from 41 (12, 75.2) nmol/L to 9.4 (2.74, 22.15) nmol/L; individual data are shown in the figure below (Fig. **5c**).

### 3.5 Adverse Reactions

Adverse events (AEs) were reported in 27.5% of patients, with headache being the most common. Influenza A and coronavirus disease-2019 (COVID-19) were present in two patients. One patient had treatment termination, and the other had delayed administration of efgartigimod. In addition, one patient experienced muscle pain, and another experienced diarrhea and nausea. An allergy occurred in one patient shortly after the injection (Table **2**). No serious AEs were observed.

## 4 DISCUSSION

Our prospective study provides evidence for the use of efgartigimod in clinical practice for new-onset AChR-gMG. Our investigation suggests that efgartigimod is clinically beneficial, offering rapid symptom control in patients with new-onset gMG, consistent with the outcomes of the ADAPT study. Early use of efgartigimod decreases the dose of corticosteroids, thereby reducing long-term side effects and improving the longitudinal prognosis. Therefore, the treatment regimen for patients with new-onset gMG may be more aggressive, which may bring more benefits and broaden the prospects for individualized MG treatment.

To date, six real-world studies have indicated the potential benefits of efgartigimod in gMG in the USA, Japan, Israel, China, and Italy. Katyal *et al*. first validated the efficacy and safety of efgartigimod for the treatment of gMG in a real-world study conducted at five centers in the United States [3]. Subsequently, Singer *et al*. found that continuous dosing of efgartigimod achieved MSE in 58.33% of patients with gMG and reduced the dose of prednisone by 53.2% [4]. Frangiamore *et al*. found that the multiple-cycle use of efgartigimod could reduce hospitalization rates, plasma exchange (plasmapheresis) or IVIg treatment rates, and ICU admission rates in patients with gMG, and improve their quality of life [5]. Recently, Fuchs *et al*. discovered two response modes of efgartigimod in patients with gMG in Israel, further guiding dosing regimens in the real world [6]. A multicenter real-world cohort study in China demonstrated the efficacy of efgartigimod in the rapid control of gMG; however, data regarding the effect of efgartigimod in patients with new-onset gMG are scarce [7]. We conducted a prospective study to explore whether efgartigimod can be used as an early active treatment for new-onset generalized myasthenia gravis (gMG) to yield better outcomes.

One area of interest in gMG treatment is the initiation of molecular therapies. Usually, patients who are refractory to common therapies, have severe side effects and contraindications to current treatments, or experience frequent myasthenia crisis (MC) are the focus of consideration for switching to monoclonal antibody therapies. However, the RINOMAX study by Fredrik *et al*. found that low-dose rituximab resulted in a higher proportion of patients with MG achieving MSE and reduced the use of rescue medications [8]. Patients with new-onset generalized myasthenia gravis (gMG) respond adequately and rapidly to rituximab compared with those with a longer disease duration, highlighting the advantage of early rituximab use in patients with MG. Therefore, we speculated that efgartigimod could be used as an early, fast-acting therapy for symptomatic improvement in new-onset generalized myasthenia gravis (gMG). Thus, efgartigimod can be used as a bridge therapy, allowing patients to gradually progress to traditional treatments that require a longer time to take effect.

Significant clinical improvement was observed in all patients at an early stage after the first cycle of efgartigimod infusion, with a mean improvement time of 0.81 weeks. Patients had greater total mean score improvements in the MG-ADL and QMG after one cycle. Additionally, 96% of the patients demonstrated an MG-ADL response compared with baseline after one cycle, which was higher than the results of the ADAPT studies (77.8%). The proportions of clinical improvement in MG-ADL scores were similar between the two subgroups (AChR antibody level <20 nmol/L *vs*. ≥20 nmol/L), suggesting that there may not be a limitation of antibody titer for the use of efgartigimod, thereby expanding the spectrum of appropriate populations. In other words, patients with lower antibody titers benefited from the use of efgartigimod. In our study, a higher proportion of patients (52%) achieved MSE, suggesting that efgartigimod is more effective during the early stages of the disease. Moreover, comparing our findings with those of the ADAPT study, the percentage of MG-ADL responders was 72% *vs*. 34.1% at 12 weeks. We also observed sustained improvement, indicating that the early use of efgartigimod may enable the attainment of MSE earlier and maintain favorable conditions. Nagane *et al*. reported that the poor response to traditional immunosuppression may be due to alterations in immunopathological conditions during long-term disease. Changes in the structure of the neuromuscular junction and immune milieu resulting from long-term MG make most patients targets for immunotherapy in an early and aggressive way [9]. Within the first two years of treatment, a positive early response indicated a benign course and improved long-term prognosis [10]. There are three possible explanations for the relatively lasting effects of efgartigimod. First, efgartigimod removes pathogenic autoantibodies and inhibits complement activation and postsynaptic membrane lysis. Second, FcRn blockade prevents the production of proinflammatory cytokines, including interleukin (IL)-12, IL-23, tumor necrosis factor, and IL-6, by neutrophils, monocytes, macrophages, and dendritic cells during innate immune interactions. Additionally, FcRn antagonists inhibit FcRn's involvement in the antigen presentation pathway, further impairing T-cell activation and thereby dampening the immune response. FcRn blockade is also involved in the upstream pathogenicity of MG, maintaining immune homeostasis through innate and adaptive immune interactions [11]. Therefore, early and aggressive treatment may lead to a better longitudinal prognosis and fine reversal of the postsynaptic membrane. Further studies are needed to confirm that the early course of the disease may determine the long-term response to treatment.

Three patients in our study with thymomatous MG (TMG) experienced recurrent symptoms and failed to achieve MSE after two cycles of infusions. Patients with TMG presented a worse response to efgartigimod in our study and required two infusion cycles, which is consistent with the study by Luo *et al*. [7]. Deletion of the autoimmune regulator in thymomas drives ‘promiscuous’ expression of peripheral tissue autoantigens (including the AChR α subunit) in thymic epithelial cells as well as a reduction or absence of thymic myoid cells in thymoma. These alterations lead to defective regulatory T-cell production in thymomas, resulting in the export of large numbers of autoreactive T cells to the periphery, which activate the immunopathological pathway and ultimately lead to the production of autoantibodies. This process typically occurs before thymectomy, allowing the peripheral immune system to maintain its pathogenic mechanisms even after thymectomy [12, 13]. Patient 4 (Fig. **4d**) was a 53-year-old male who presented with ptosis, dysphagia, and extremity weakness. With an initial MG-ADL score of 17, prednisone (30-60 mg qd) and pyridostigmine (60-120 mg tid) were continuously administered. The first administration of efgartigimod was completed with only two infusions. Subsequently, the patient developed MC and infection, requiring intensive care and IVIg infusion. The ADL score was 12 at the start of the second cycle and decreased to 8 after four infusions. The patient underwent thymectomy later, but the ADL score remained at 13; therefore, he was referred for B-cell depletion therapy as the next therapy regimen. The patient was refractory to conventional immunotherapies and did not achieve stable symptom control after two cycles of efgartigimod and other treatments. The poor prognosis of patients with TMG treated with efgartigimod has led clinicians to be vigilant about multiple cycles in such cases.

All the patients in this study were able to reduce their daily steroid doses. The mean daily dose of prednisone was decreased by 10.73 mg per day. Patients with new-onset MG showed a significant reduction in oral prednisone dose, which was indicative of remarkable early improvement. Our study demonstrates that efgartigimod exerts a steroid-sparing effect that attenuates the occurrence of AEs. Conventional regimens of prednisone in MG therapy are typically initiated at small doses and slowly ramped up, which adds to the burden of steroid-related AEs. Early fast-acting therapy may allow earlier achievement of MSE while reducing the steroid dosage and duration. Based on our results, efgartigimod may be recommended as a fast-acting treatment for new-onset AChR-gMG to induce MSE at lower doses of oral corticosteroids.

The treatment was well tolerated, and a few mild AEs were reported. One patient discontinued treatment due to COVID-19. Other common AEs included diarrhea, mild allergic reactions, headache, and muscle pain. However, the side effects were mild, and the use of efgartigimod was not discontinued. No significant changes were observed in albumin or other Ig levels after efgartigimod treatment.

Our prospective analysis has several limitations. First, it was a single-center, real-world study, and as such, it only evaluated a small population of patients with MG. Moreover, our study had the limitations of being an uncontrolled trial and having shorter follow-up periods. A longer clinical follow-up is needed to clarify the long-term prognosis and the timing of retreatment with efgartigimod. Although patients with MuSK, LRP4, and triple-seronegative MG were excluded from our study, they should be given more consideration in subsequent studies. Further studies are urgently needed to determine the optimal timing and criteria for retreatment before MG exacerbation.

## CONCLUSION

Our study demonstrates the effectiveness and safety of efgartigimod in treating patients with new-onset AChR-gMG. Data from the study indicated that 52% of patients achieved MSE and 96% of patients achieved MG-ADL response after one cycle, suggesting that early administration of efgartigimod could lead to rapid control of MG symptoms. Furthermore, a significant reduction in the daily prednisone dose was observed, indicating that efgartigimod may mitigate the complications associated with steroid use. In summary, efgartigimod has the potential to serve as a bridge treatment option, facilitating the steady transition to long-term conventional immunosuppressive therapy. These findings may expand the therapeutic range and inform clinical practice. The optimal timing, duration, and retreatment criteria for efgartigimod in the treatment of MG remain unclear. These aspects warrant further investigation, and larger randomized controlled trials are necessary to confirm these findings and explore the role of efgartigimod as a therapeutic bridge for new-onset gMG.

## AUTHORS’ CONTRIBUTIONS

The authors confirm their contribution to the paper as follows: ZRX had full access to all the data in the study and takes full responsibility for the integrity and accuracy of the data and the data analysis. The authors confirm their contribution to the paper as follows: study conception and design: ZRX; data collection: MC, SJY; data analysis: ZY; methodology: SJY; investigation: MC, SJY, ZY; draft manuscript: MC, SJY, ZY, ZRX. All authors reviewed the results and approved the final version of the manuscript.

## Figures and Tables

**Table 1:** Baseline features of patients enrolled, n = 29. SD, standard deviation.

**Clinical Variates**	**Mean ± SD (Range) or ** **Percentage (%)**
Sex (%)	-
Male	15 (52%)
Female	14 (48%)
Age (years old)	62.24 ± 11.37
Thymoma (%)	4 (14%)
**MGFA Classification at Efgartigimod Initiation**	
II	2 (7%)
III	20 (69%)
IV	7 (24%)
**Clinical Classification (%)**	
EOMG	3 (10%)
LOMG	26 (90%)
Mean baseline QMG score	13.52 ± 3.64
Mean baseline MG-ADL score	10.38 ± 2.71
**Treatment at Efgartigimod Initiation (%)**	
Pyridostigmine	28 (97%)
Prednisone	20 (69%)
Tacrolimus	7 (24%)

**Note**: MGFA, Myasthenia Gravis Foundation of America. EOMG, Age of onset < 50 years. LOMG, Age of onset ≥ 50 years. QMG, Quantitative Myasthenia Gravis. MG-ADL, Myasthenia Gravis Activities of Daily Living.

**Table 2:** Adverse events observed in the study.

**Adverse Events**	**Percentage (%)**
Headache	2 (7%)
Allergy	1 (3.4%)
Muscle pain	1 (3.4%)
Influenza virus infection	2 (7%)
Diarrhea	1 (3.4%)
Nausea	1 (3.4%)

## Data Availability

All the data and supporting information are provided within the article.

## LIST OF ABBREVIATIONS

AChR: Acetylcholine Receptor
AEs: Adverse Events
COVID-19: Coronavirus Disease-2019
IgG: Immunoglobulin G
LRP4: Lipoprotein Receptor-related Protein 4
MuSK: Muscle-specific Tyrosine Kinase
MC: Myasthenia Crisis
MG: Myasthenia Gravis
